# Tertiary Syphilis in the Nose and Pharynx

**Published:** 1901-02-02

**Authors:** 


### TERTIARY SYPHILIS IN THE NOSE AND PHARYNX.

Writing on the treatment of tertiary syphilitic lesions in the nose and pharynx, Mr. Charles Parker[1] says that very often when iodide of potassium alone, even in very big doses is inefficient, the affection will very readily yield to iodide of potassium and mercury combined, the best way of administering the mercury in these cases being by inunction. He also gives a caution in regard to giving iodide where there is much stenosis of the larynx, for the first effect of the drug is often to cause rapid œdema of the laryngeal mucous membrane, and thus greatly to increase the obstruction. If, therefore, there is much obstruction to start with, iodide should only be given if the patient is within easy reach of help, for tracheotomy might become urgently necessary. In tertiary syphilis of the nose, after the removal of all crusts and necrotic tissue, and the scraping away of granulations with a curette, the passages must be kept clean with an alkaline lotion. In obstinate cases, after cleansing, heated calomel should be insufflated, and the nose should be packed with double cyanide gauze. Packing has a marked effect in preventing the formation of crusts, and in keeping the mucous membrane clean and moist. The patient can change the gauze himself, and this should be done daily. In tertiary ulceration of the pharynx the surface of the ulcer should be cleansed, and may then be insufflated with iodoform, or in some cases with heated calomel. In many cases in which there is much pain, the insufflation of orthoform will be attended with results most grateful to the patient. One insufflation will often relieve pain for as long as twenty-four hours, so that daily application will keep the patient quite comfortable. A most useful gargle for cleansing the throat and relieving pain is one composed of chlorate of potash, 10 grains; lotio nigra, half an ounce; water to one ounce. As to adhesions, let them alone unless there are urgent symptoms, in which case stretch the opening by the regular passage of dilating instruments of gradually increasing size.

[1] Lancet, Jan. 26.

